# A New Photographic Reproduction Method Based on Feature Fusion and Virtual Combined Histogram Equalization

**DOI:** 10.3390/s21186038

**Published:** 2021-09-09

**Authors:** Yu-Hsiu Lin, Kai-Lung Hua, Yung-Yao Chen, I-Ying Chen, Yun-Chen Tsai

**Affiliations:** 1 Graduate Institute of Automation Technology, National Taipei University of Technology, Taipei 106, Taiwan; yhlin@ntut.edu.tw (Y.-H.L.); t105618031@ntut.edu.tw (I.-Y.C.); t106618009@ntut.edu.tw (Y.-C.T.); 2 Department of Computer Science and Information Engineering, National Taiwan University of Science and Technology, Taipei 106, Taiwan; hua@mail.ntust.edu.tw; 3 Department of Electronic and Computer Engineering, National Taiwan University of Science and Technology, Taipei 106, Taiwan

**Keywords:** photographic reproduction, vision sensing technique, feature fusion, human visual system, virtual combined histogram, histogram equalization

## Abstract

A desirable photographic reproduction method should have the ability to compress high-dynamic-range images to low-dynamic-range displays that faithfully preserve all visual information. However, during the compression process, most reproduction methods face challenges in striking a balance between maintaining global contrast and retaining majority of local details in a real-world scene. To address this problem, this study proposes a new photographic reproduction method that can smoothly take global and local features into account. First, a highlight/shadow region detection scheme is used to obtain prior information to generate a weight map. Second, a mutually hybrid histogram analysis is performed to extract global/local features in parallel. Third, we propose a feature fusion scheme to construct the virtual combined histogram, which is achieved by adaptively fusing global/local features through the use of Gaussian mixtures according to the weight map. Finally, the virtual combined histogram is used to formulate the pixel-wise mapping function. As both global and local features are simultaneously considered, the output image has a natural and visually pleasing appearance. The experimental results demonstrated the effectiveness of the proposed method and the superiority over other seven state-of-the-art methods.

## 1. Introduction

In the real world, the luminance intensity of environmental scenes has a very wide range. From glimmer starlight to blazing sunlight, the luminance variation could span over ten orders of magnitude. The human visual system (HVS) has an outstanding ability to adapt and perceive about 5~6 orders of magnitude. Previously, most consumer cameras can only capture nearly 2~3 orders of luminance variation. Due to the limited dynamic range, the captured image severely suffers from detail loss, especially in highlight and shadow regions [1].

With advancements in optical sensing, high dynamic range (HDR) sensors that can capture the entire luminance range of a real-world scene have been developed [2]. For example, some latest high-end digital single-lens reflex cameras, or some devised sensors including multiple sensor elements with different exposure levels, are able to capture entire details of both dark and bright parts of the scene simultaneously. Although the cost of such HDR sensors is high, the captured HDR images can contain a larger bit depth of the image than 24-bit depth per pixel. Typically, HDR images are stored in floating point and require 32-bit depth per pixel.

Despite the increasing availability of HDR images, presenting HDR scenes on traditional low dynamic range (LDR) or standard dynamic range (SDR) displays remains problematic because an SDR display can only display 256 brightness levels. The high cost and the under-developing display technology remain major obstacles to the mass production of HDR display devices. To solve this problem, the photographic reproduction method becomes an essential technique and has been a prominent subject in the field of image sensing research or the image-based applications [3].

## 2. Related Work

A photographic reproduction method should not only provide contrast adjustment but also the preservation of the luminance, details, and even the vividness of the colors of the original image. According to their modeling characteristics, most photographic reproduction methods are usually divided into the following three categories. First, the global-based photographic reproduction methods perform the same mapping function for all image pixels based on the global features of the input HDR image. In other words, an input pixel value produces a specific output value, regardless of its position. Both linear mapping functions and different nonlinear mapping functions are used to mimic the HVS. Reinhard and Devlin [4] proposed a global-based reproduction method using electrophysiology and the photoreceptor model that reflects human perception. It is a fast algorithm; however, the detail preserving is not considered. Mantiuk et al. [5] presented a piece-wise linear reproduction method that minimizes visible distortions by considering the penalty using the HVS contrast perception model. To reproduce optimal scene-referred images on a range of display devices, their method can adjust the image content by considering the display characteristics and surrounding illumination. However, the local operation, such as sharpening, is not considered. In [6], Kim et al. proposed integrating a modified weighted least square filter with mapping, which can preserve detail and maintain the global contrast through the competitive learning neural network. Furthermore, the color shift issue is solved by utilizing the Helmholtz–Kohlrausch effect in the light correction stage. Gommelet et al. [7] designed a global-based reproduction method to address the optimal rate-distortion problem, which typically occurs in the reconstruction of an HDR signal. In [7], a novel distortion model was built that takes the image gradient into account. Khan et al. [8] proposed a reproduction method that uses the features of HVS and the threshold versus intensity curve to adjust the individual bin-widths of the input image histogram. A global-based reproduction function is built using the modified histogram; however, the local information of an image is not used during the reproduction.

The overall pros of the global-based photographic reproduction methods are the capability to preserve the global contrast of the original images, and in addition, the computational complexity is low. However, the global compression of the dynamic range is typically accompanied by the suppression of local contrast, which is the inevitable con of the global-based methods. Moreover, especially for the scenes with a large difference in brightness, the bright and dark regions are the most severely sacrificed by detail loss because, compared to the entire dynamic range, the local intensity variation in such regions are almost ignorable.

To overcome the shortcomings of global-based methods, local-based photographic reproduction methods are proposed. For the local-based reproduction methods, different reproduction functions are designed to each pixel based on the pixel position and its surrounding pixels. Different pixel positions can share the same intensity but may relate to different reproduced values. Ahn et al. [9] proposed a local-based reproduction method, which utilizes center/surround retinex theory to adapt the local contrast. This work demonstrates superior contrast enhancement in the scenes with low log-average luminance. Nevertheless, in [9], sometimes over-enhancement occurs in the details. Tan et al. [10] proposed a halo-free reproduction method, which applies using an L0 smoothing filter to mimic the adaptability of the HVS mechanism. Cyriac et al. [11] presented a two-stage reproduction method, where the first stage is a simple gamma curve for global compression, and the second stage is a local-based reproduction scheme using psychophysical models for the visual cortex. However, the local-based processing in the second stage tends to degrade the overall naturalness. Croci et al. [12] proposed a reproduction method to reproduce the HDR video, where a tone-curve-space alternative is used as a substitute for the temporal per-pixel coherency to increase the computational efficiency. Li et al. [13] presented a clustering-based content and color adaptive reproduction method, which divides the input image into patches. By analyzing the local content information (e.g., patch mean, color variation, and color structure), the patches are formed into clusters, and the tone mapping is performed via a more compact domain. However, the patch-based processing tends to ignore the image information as a whole.

Due to considering local features, the typical pro of the local-based photographic reproduction methods is to provide more local details in dark and bright regions compared with global-based reproduction methods. Therefore, the local-based methods are suitable to the scenes where a large brightness difference exists. However, they are vulnerable to artifacts such as halo effects and block effects, which cause an unnatural overall appearance. Moreover, the global contrast decreases because of the lack of global features.

As both global- and local-based photographic reproduction methods have some drawbacks, some researchers have proposed decomposition-based photographic reproduction methods, which use decomposition techniques to obtain large-scale image structures (i.e., base layers) and small-scale image textures (i.e., detail layers), and thus, global-based (and local-based) approaches can be used for specific layers accordingly. Gu et al. [14] designed a local edge-preserving filter that has a locally adaptive property. Based on the filter, a retinex-based approach is presented, where the image is decomposed into one base layer and three detail layers for the reproduction manipulation. Barai et al. [15] proposed an HVS-inspired reproduction method, where the saliency map information is fed into the guided filter for image decomposition. Then, global compression and detail enhancement are performed in the base layer and the detail layer, respectively. Mezeni and Saranovac [16] presented an enhanced reproduction method, which decomposes the image into base/detail layers. Then, the base layer is scaled partially in the linear domain and partially in the logarithmic domain, and a detail enhancement is performed in the dark areas of the detail layer. However, when generating the output image, it is hard to fuse individual layers suitably. Liang et al. [17] presented a hybrid layer decomposition model for photographic reproduction, where a sparsity term is used to model the piecewise smoothness of the base layer, and the other sparsity term with a structural prior is used to model the piecewise constant effect of the detail layer. Miao et al. [18] presented a macro–micro-modeling reproduction method, in which multi-layer decomposition is utilized from the perspective of the micro model, and content-based global compression is utilized from the perspective of the macro model. The representative pro of the decomposition-based photographic reproduction methods is the flexibility to deal with different base and detail layers separately. However, the con of such a method is the difficulty of blending individual layers smoothly. That is, some blurs tend to occur in the final layer fusion process.

**Figure 1:**
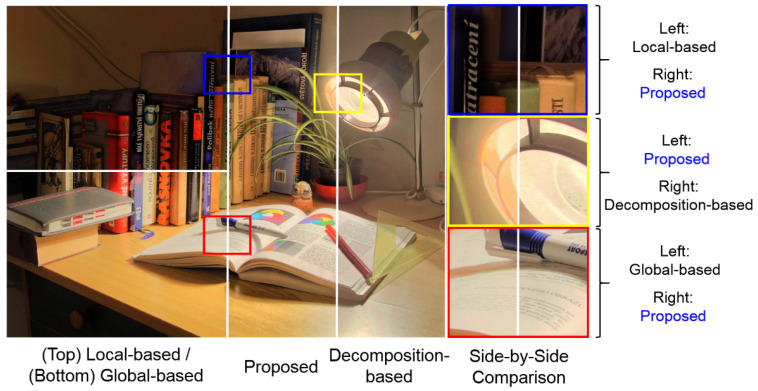
A rough comparison among a global-based reproduction method [8] (**bottom left**), a local-based reproduction method [9] (**top left**), a decomposition-based reproduction method [18] (**right**), and our proposed method (**middle**). This example shows that our method inherits the advantages of both global- and local-based reproduction method, while avoiding the unnaturalness issue of decomposition-based reproduction method (due to processing different layers separately.

To exemplify the superiority of this work, Figure 1 shows a visual comparison among the global-based, local-based, decomposition-based methods, and our proposed method. In view of the abovementioned shortcomings of the global-based, local-based, and decomposition-based methods, this paper presents a new photographic reproduction method, which has the following three main advantages:

- We propose using a hybrid histogram analysis scheme to extract mutually compatible global/local features in parallel, and a feature fusion scheme to construct the virtual combined histogram, which allows us to inherit the superiority of the global-based (and the local-based) methods smoothly.
- Instead of performing late fusion (i.e., finally fusing all the processed layers as the decomposition-based methods do), the proposed virtual combined histogram equalization scheme can fuse global/local features in an earlier stage, which increases the naturalness of the output image.
- Owning to the difference between the dark/bright regions and normal-luminance regions, we propose using the weight map to adaptively modify the weights locally in the feature fusion.

## 3. Proposed Approach

### 3.1. Pre-Processing for the Highlight/Shadow Detection

**Figure 2:**
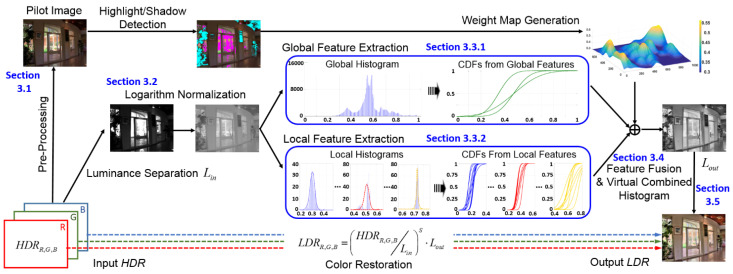
Flowchart of the proposed method, where the blue bold words indicate the section numbers.

Figure 2 shows the overall framework of this study. The proposed method is designed due to the strategy of improving the visibility of highlight and shadow areas, while maintaining the global naturalness of the original image. In the pre-processing stage, a quick photographic reproduction method [19] is applied to the original HDR signal to obtain a pilot image ($I^{Pilot}$), which is a preliminary reproduced result with a simple global compression. Although the pilot image might suffer from detail loss locally, it is good enough for us to distinguish the dark/bright regions from the normal-luminance regions.

Subsequently, we modify the work of [20] for highlight/shadow detection as follows. First, a specular-free image ($I^{SF}$) is defined as follows:

(1) $$I_{c}^{SF}\left(i,j\right)=I_{c}^{Pilot}\left(i,j\right)-I^{Dark}\left(i,j\right),$$

where the subscript c∈R,G,B indicates one of the RGB color channels, and the dark channel ($I^{Dark}$) is defined as follows:

(2) $$I^{Dark}\left(i,j\right)=\underset{c\in R,G,B}{\min}\;I_{c}^{Pilot}\left(i,j\right)\;.\;$$

As $I^{SF}$ is obtained by subtracting the minimum of RGB values from $I^{Pilot}$, at least one of the three channels in $I^{SF}$ equals zero at each pixel position. Then, the modified specular-free (MSF) image is obtained by adding the average of the dark channel image to the specular-free image as follows:

(3) $$I_{c}^{MSF}\left(i,j\right)={\bar{I}}^{Dark}+I_{c}^{SF}\left(i,j\right),$$

**Figure 3:**
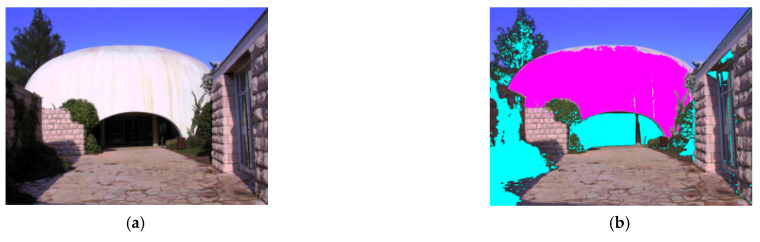
An example of highlight/shadow detection. (**a**) The pilot image (pre-processed using the method of [19]). (**b**) Detection results from the proposed method, where the pink and cyan areas indicate highlights and shadows, respectively.

In [20], the difference between the MSF image and the pilot image can be used to detect the highlight regions in the image. With this feature, we find that if we multiply a correction parameter (θ) with the threshold and compare it with the pilot image, we can also detect shadow regions. Therefore, the proposed highlight/shadow detection scheme can be expressed as follows:

(4) $$\mathrm{pixel}\;\epsilon \;\{\begin{array}{cc} \mathrm{highlight}, & \mathrm{if}\;\delta_{c}\left(i,j\right)>thr\;\mathrm{for}\;\mathrm{all}\;c\\ \mathrm{shadow}, & \;\;\;\;\;\;\;\;\;\;\;\mathrm{if}\;I_{c}^{Pilot}\left(i,j\right)<\theta \cdot thr\;\mathrm{for}\;\mathrm{all}\;c\\ \mathrm{midtone}, & \mathrm{otherwise} \end{array}\;.\;$$

where $\delta_{c}=I_{c}^{Pilot}-I_{c}^{MSF}$, θ=0.8 is an empirical value (from our experiments, 0.75≤θ≤0.85 would produce accurate detection result), and the threshold value (thr) is obtained by applying the Otsu method in the pilot image. The Otsu method is an automatic way of creating binarization in image processing, and we find that it is suitable to determine the threshold in Equation (4). Figure 3 shows an example of the highlight/shadow detection results, which will be used as the estimate of the steering weight coefficients in the feature fusion stage (described later in Section 3.4).

### 3.2. Luminance Separation and Initial Logarithmically Normalization

As luminance information is mainly affected by the dynamic range, distinguishing luminance and chrominance from the original HDR signal is a common approach in photographic reproduction. In this study, luminance information was extracted by converting from RGB color space to CIE XYZ color space through the ITU-R BT.709 standard.

(5) $$L_{in}=0.2126\cdot I_{R}^{H}+0.7152\cdot I_{G}^{H}+0.0722\cdot I_{B}^{H},\;$$

where $I^{H}$ indicates the input HDR signal and $L_{in}$ indicates the corresponding luminance, which contains no chromatic information.

For different scenes, their dynamic range may vary quite greatly. To avoid the inconsistent dynamic range issue, the logarithmic function is a typical process to compress the luminance domain according to the following Weber–Fechner theory:

(6) $$L_{\log}\left(i,j\right)=\log_{10}\left(L_{in}\left(i,j\right)+10^{-6}\right),$$

where $10^{-6}$ is added to avoid the singularity error occurring as the input pixel luminance equals zero. Furthermore, to match the property that perceived brightness is proportional to the logarithm of the actual luminance intensity, its logarithmically normalized value can be expressed as follows:

(7) $$L_{\log\_n}\left(i,j\right)=\frac{L_{\log}\left(i,j\right)-\min\left(L_{\log}\left(i,j\right)\right)}{\max\left(L_{\log}\left(i,j\right)\right)-\min\left(L_{\log}\left(i,j\right)\right)}\;,\;$$

where $\max\left(L_{\log}\left(i,j\right)\right)$ and $\min\left(L_{\log}\left(i,j\right)\right)$ represent the maximum and minimum values of $L_{\log}\left(i,j\right)$, respectively. To adapt various lighting conditions, the normalized logarithmic luminance value ($L_{\log\_n}\left(i,j\right)$), which always ranges between 0 and 1, are analyzed in the following steps.

### 3.3. Feature Extraction through Mutually Hybrid Histogram Analysis

The main challenge in photographic reproduction is to preserve both the global and local features of the original image, i.e., maintaining both the entire luminance balance and local detail information. In this study, the abovementioned features are neither the feature points used in computer vision, nor the feature vectors used in machine learning. The feature represents the general property of the entire image (i.e., global feature) or of individual local regions (i.e., local features) that are needed in the proposed photographic reproduction procedure.

Some proposed reproduction methods perform the global-based (and local-based) processes separately; in other words, they first apply a global luminance adaption and then perform local detail enhancement. However, we argue that this type of two-step strategy may not be the optimal solution because the goals of these two steps are inherently conflicting: one is to enhance the global features, and the other one is to enhance the local features.

**Figure 4:**
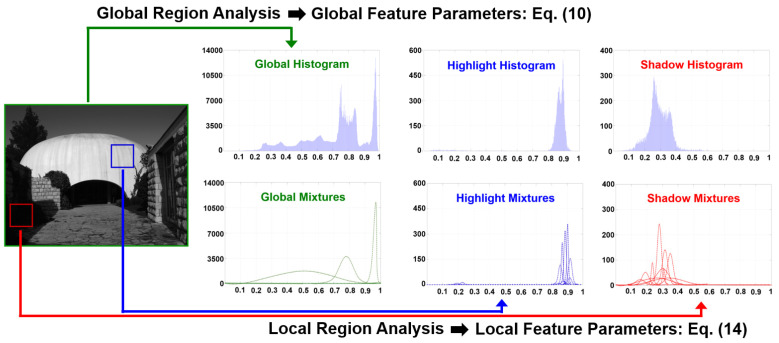
Proposed mutually hybrid histogram analysis approach. We analyze the global and local histograms simultaneously using different statistical methods, i.e., Gaussian mixture model for the former and stratified sampling for the latter. Although different statistical methods are applied, we aim to extract the mutually compatible features to form a virtual combined histogram (introduced later in Section 3.4).

As shown in Figure 4, we propose a parallel framework to simultaneously analyze the global histogram (constructed by the entire image) and local histogram (constructed by individual local image patches). The underlying concept of the proposed mutually hybrid histogram analysis is to extract the mutually compatible features from two statistical approaches.

#### 3.3.1. Global Region Analysis and Global Feature Extraction

In global region analysis, the logarithmically normalized luminance plane is first transformed into a global histogram of K levels with equal bin width, where K is empirically set as one thousand. When divided by the total number of pixels in the image, the global histogram $h^{G}\left(x_{k}\right)$ can be viewed as a probability density function of pixels. A parametric statistical method called the Gaussian mixture model (GMM) can then be used to structure $h^{G}\left(x_{k}\right)$ as a weighted summation of three Gaussian functions as follows:

(8) $$h^{G}\left(x_{k}\right)=\overset{3}{\underset{n=1}{\sum }}\alpha_{n}^{G}\cdot g\left(x_{k},\mu_{n}^{G},\sigma_{n}^{G}\right),$$

(9) $$g\left(x_{k},\mu_{n}^{G},\sigma_{n}^{G}\right)=\frac{1}{\sigma_{n}^{G}\sqrt{2\pi }}\exp\left[\frac{-{\left(x_{k}-\mu_{n}^{G}\right)}^{2}}{2{\left(\sigma_{n}^{G}\right)}^{2}}\right]\;,$$

where $\left\{x_{k},k=0,1,\ldots ,K-1\right\}$ indicates the quantized reproduced levels of $L_{\log\_n}$, and $\alpha_{n}^{G},n=1,2,3$ is the weight of the n-th Gaussian function. The reason for using three Gaussian functions to approximate $h^{G}\left(x_{k}\right)$ is because in photographic reproduction, we normally concern the following three main parts: the highlight area, midtone area, and shadow area. From Equation (8), we refer to the global feature set as the following:

(10) $$\theta^{G}=\left\{\alpha_{n}^{G},\mu_{n}^{G},\sigma_{n}^{G}|n=1,2,3\right\}\;\;.\;$$

The expectation-maximization (EM) algorithm [21] was adopted to solve the GMM estimation problem, which is used to find the maximum likelihood estimates of parameters in the statistical models involving unobserved latent variables. In this study, the likelihood function is defined as follows:

(11) $$\mathrm{Likelihood}\left(\theta^{G}\right)=\ln\left[\overset{K-1}{\underset{K=0}{\prod }}h^{G}\left(x_{k}\right)\right]=\overset{K-1}{\underset{K=0}{\prod }}\ln h^{G}\left(x_{k}\right)\;.\;$$

To efficiently find the optimal $\theta^{G}$, the derivatives of the log-likelihood with respect to the initial $\alpha_{n}^{G}$,$\;\mu_{n}^{G}$, and $\sigma_{n}^{G}$ are, respectively, set as zero (i.e., the expectation step), which yields a new parameter set of GMM (i.e., the maximization step). The EM algorithm iteratively switches between the expectation step and the maximization step until it converges (Please refer to [21] for the details of EM).

#### 3.3.2. Local Region Analysis and Local Feature Extraction

**Figure 5:**
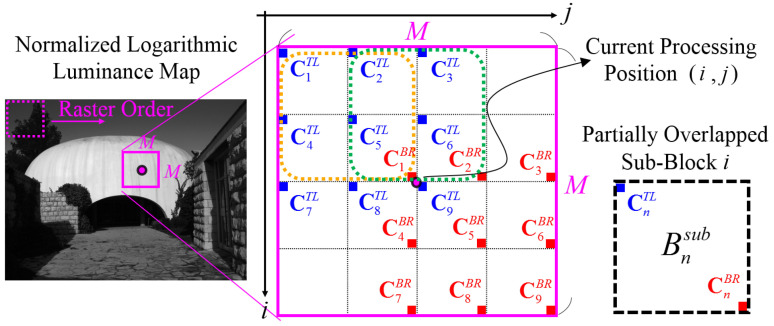
Local region analysis, where the orange and the green squares, respectively, indicate the first and the second partially overlapped subblocks of a local region (the pink square with size of M×M). At each processing position, local features are extracted by the statistical analysis of nine subblocks defined in Equations (12) and (13), and its simplification is achieved by utilizing the summed-area table defined in Equations (16) and (17).

In local region analysis, a sliding window scheme is adopted to visit each individual local region in raster scan order. Figure 5 illustrates the local region analysis, where a local region is of size M×M (M=129 as the default) and is centered at the current processing position (i,j). Each local region is first divided into sixteen units with a size of 32 × 32 pixels, and 2 × 2 units constitute a partially overlapped subblock, e.g., the orange (or the green) square shown in Figure 5. 

With consideration of the estimation accuracy and computation cost, each local region is subsampled into nine partially overlapping subblocks ($B_{n}^{sub}$) that correspond to the two corner sets. First, the top-left (TL) corner set can be expressed by the following:

(12) $$\left\{C_{n}^{TL},n=1,2,\ldots ,9\right\}\;.$$

where $C_{1}^{TL}=\left(i-\lfloor M/2\rfloor ,\;j-\lfloor M/2\rfloor \right)$, $C_{2}^{TL}=\left(i-\lfloor M/2\rfloor ,\;j-\lfloor M/4\rfloor \right)$, $C_{3}^{TL}=\left(i-\lfloor M/2\rfloor ,\;j+1\right)$, $C_{4}^{TL}=\left(i-\lfloor M/4\rfloor ,\;j-\lfloor M/2\rfloor \right)$, $C_{5}^{TL}=\left(i-\lfloor M/4\rfloor ,\;j-\lfloor M/4\rfloor \right)$, $C_{6}^{TL}=\left(i-\lfloor M/4\rfloor ,\;j+1\right)$, $C_{7}^{TL}=\left(i+1,\;j-\lfloor M/2\rfloor \right)$, $C_{8}^{TL}=\left(i+1,\;j-\lfloor M/4\rfloor \right)$, and $C_{9}^{TL}=\left(i+1,\;j+1\right)$. Second, the bottom-right (BR) corner set can be expressed by the following:

(13) $$\left\{C_{n}^{BR},n=1,2,\ldots ,9\right\}\;.$$

where $C_{1}^{BR}=\left(i-1,\;j-1\right)$, $C_{2}^{BR}=\left(i-1,\;j+\lfloor M/4\rfloor \right)$, $C_{3}^{BR}=\left(i-1,\;j+\lfloor M/2\rfloor \right)$, $C_{4}^{BR}=\left(i+\lfloor M/4\rfloor ,\;j-1\right)$, $C_{5}^{BR}=\left(i+\lfloor M/4\rfloor ,\;j+\lfloor M/4\rfloor \right)$, $C_{6}^{BR}=\left(i+\lfloor M/4\rfloor ,\;j+\lfloor M/2\rfloor \right)$, $C_{7}^{BR}=\left(i+\lfloor M/2\rfloor ,\;j-1\right)$, $C_{8}^{BR}=\left(i+\lfloor M/2\rfloor ,\;j+\lfloor M/4\rfloor \right)$, and $C_{9}^{BR}=\left(i+\lfloor M/2\rfloor ,\;j+\lfloor M/2\rfloor \right)$. Each pair of $\left(C_{n}^{TL},C_{n}^{BR}\right)$ specifies the n-th subblock. To generate mutually compatible features (compatible to the global features) similar to Equation (8), this subsection aims to simulate each local histogram $h^{L}\left(x_{k}\right)$ as a set of nine Gaussian functions $g\left(x_{k},\mu_{n}^{L},\sigma_{n}^{L}\right)$ and to find the local feature set as the following:

(14) $$\theta^{L}=\left\{\alpha_{n}^{L},\mu_{n}^{L},\sigma_{n}^{L}|n=1,\ldots ,9\right\}\;.\;$$

Instead of using GMM, we adopt another statistical method called stratified sampling, in which the entire block is divided into homogeneous subblocks (defined as strata). The reason for partially overlapping is to avoid image artifacts such as the blocking effect and the halo effect. The distribution of each subblock is intentionally simulated as a Gaussian function, where the subblock mean and the subblock standard deviation are treated as the corresponding $\mu_{n}^{L}$ and $\sigma_{n}^{L}$ in Equation (14), respectively. In addition, a spatial kernel (**K**) is used to weight the spatial correlation as follows:

(15) $$K=\left\lfloor \begin{array}{ccc} \alpha_{1}^{L} & \alpha_{2}^{L} & \alpha_{3}^{L}\\ \alpha_{4}^{L} & \alpha_{5}^{L} & \alpha_{6}^{L}\\ \alpha_{7}^{L} & \alpha_{8}^{L} & \alpha_{9}^{L} \end{array}\right\rfloor =\frac{1}{51}\left\lfloor \begin{array}{ccc} 5 & 6 & 5\\ 6 & 7 & 6\\ 5 & 6 & 5 \end{array}\right\rfloor \;.$$

Inspired by [22], we adopted a summed-area table approach [23] to reduce the computation complexity of local region analysis as follows. First, the summed-area table ($T_{SA}$) was generated by calculating the sum of all the pixels above and to the left of the current position as the following:

(16) $$T_{SA}\left(i,j\right)=\underset{i^{\prime }\leq i,j^{\prime }\leq j}{\sum }L_{\log\_n}\left(i^{\prime },j^{\prime }\right),$$

Similar to Equation (16), the square summed-area table ($T_{SA}^{2}$) was generated by calculating the sum of all pixel squares as the following:

(17) $$T_{SA}^{2}\left(i,j\right)=\underset{i^{\prime }\leq i,j^{\prime }\leq j}{\sum }L_{\log\_n}^{2}\left(i^{\prime },j^{\prime }\right)$$

Notably, both $T_{SA}$ and $T_{SA}^{2}$ could be efficiently computed through a one-pass procedure over the image by the following:

(18) $$T_{SA}^{p}\left(i,j\right)=L_{\log\_n}^{p}\left(i,j\right)+T_{SA}^{p}\left(i,j-1\right)\;\;\;\;\;\;\;\;\;\;\;\;\;\;\;\;\;\;\;\;\;\;\;\;\;\;\;\;\;\;\;\;\;\;\;\;\;\;\;+T_{SA}^{p}\left(i-1,j\right)-T_{SA}^{p}\left(i-1,j-1\right),$$

where p=1 and 2.

Once the two summed-area tables were generated, the mean and standard deviation of each subblock could be quickly obtained by looking up $T_{SA}$ and $T_{SA}^{2}$ because of the following closed-form solutions:

(19) $$\left(\mathrm{Mean}\right)\;\mu =\frac{1}{N}\lfloor T_{SA}\left(i_{1},j_{1}\right)+T_{SA}\left(i_{0}-1,j_{0}-1\right)\;\;\;\;\;\;\;\;\;\;\;\;\;\;\;\;\;\;\;\;\;\;\;\;\;\;\;\;\;-T_{SA}\left(i_{0}-1,j_{1}\right)-T_{SA}\left(i_{0},j_{1}-1\right)\rfloor ,$$

(20) $$\left(\mathrm{Standard}\;\mathrm{Deviation}\right)\;\sigma =\sqrt{\frac{1}{N}\left\lfloor S-\frac{\mu^{2}}{N}\right\rfloor ,}$$

where *N* is the number of pixels in the subblock, and $S=T_{SA}^{2}\left(i_{1},j_{1}\right)+T_{SA}^{2}\left(i_{0}-1,j_{0}-1\right)-T_{SA}^{2}\left(i_{0}-1,j_{1}\right)-T_{SA}^{2}\left(i_{0},j_{1}-1\right)$. The four positions $\left(i_{0},j_{0}\right),\;\left(i_{0},j_{1}\right),\;\left(i_{1},j_{0}\right),$ and $\left(i_{1},j_{1}\right)$ indicate the top-left, the top-right, the bottom-left, and the bottom-left corners of the subblock, respectively.

### 3.4. Virtual Combined Histogram Construction Based on Feature Fusion

Histogram equalization (HE) is a well-known method that by analyzing the histogram, pixel intensities can be arranged for enhancing the global contrast while maintaining image details by pursuing maximum entropy. As shown in the bottom row of Figure 4, both the global histogram and the highlight/shadow local histogram can be approximated (or characterized) as Gaussian mixtures. In this study, we propose a virtual combined histogram construction scheme based on nominally fusing the local/global Gaussian mixtures as follows.

First, considering that there is minor detail loss in the normal-luminance regions and more detail loss in the under-luminance (or over-luminance) regions during the reproduction process, the highlight/shadow detection result of Equation (4) is adopted to generate a binary map, where the highlight/shadow pixels are recorded as “1”, and the midtone pixels are recorded as “0”. A weight map function ($\tau_{i,j}$) is generated by convolving the binary map with a Gaussian low-pass filter (the Matlab inbuilt imgaussfilt function) to smooth the weighting difference. By doing so, we aim to make greater use of the local features (i.e., increase the weight map value in bright/dark regions) because the details of such regions are generally vulnerable to loss. The weight map function varies in different pixel positions because the weighting of local features should be region independent. Therefore, a virtual combined histogram is constructed by fusing global and local features through the following:

(21) $$h_{i,j}^{Comb}\left(x_{k}\right)=\left(\omega_{1}-\tau_{i,j}\right)\cdot h^{G}\left(x_{k}\right)+\left(\omega_{2}+\tau_{i,j}\right)\cdot h_{i,j}^{L}\left(x_{k}\right),$$

where the subscript (i,j) indicate the pixel position, $\omega_{1}$ and $\omega_{2}$ represent the initial fusion weights (we set $\omega_{1}=0.4$ and $\omega_{2}=0.6$ empirically), and $h^{G}\left(x_{k}\right)\;$and $h_{i,j}^{L}\left(x_{k}\right)$ indicate the global and the local Gaussian mixtures, respectively. Moreover, we set an upper bound to constrain the maximum $\tau_{i,j}$ value as 0.2. That is, the minimum weight to the global Gaussian mixtures in Equation (21) is guaranteed to be 0.2 to preserve the overall naturalness.

### 3.5. Luminance Modification and Color Recovery

Through the virtual combined histogram, a look-up table is generated in the traditional HE manner with linear interpolation. That is, the output luminance plane was modified by the following:

(22) $$L_{out}\left(i,j\right)=\min\left(L_{out}\right)+\left(\max\left(L_{out}\right)-\min\left(L_{out}\right)\right)\cdot {\mathrm{CDF}}_{i,j}\left(x_{k}\right),$$

where $L_{out}$ is the adjusted luminance and ${\mathrm{CDF}}_{i,j}\left(x_{k}\right)$ is the Cumulative Distribution Function (CDF), which corresponds to the virtual combined histogram in Equation (21).

Overall, the pixel-wise modification function was controlled by manipulating both the global and local features through the virtual combined histogram. As each combined histogram was a summation of weighted Gaussian functions, the property of the Gauss error function was used to simplify the calculation by using the following:

(23) $$\Phi \left(x_{k}\;|\;\mu ,\sigma \right)=\frac{1}{2}+\frac{1}{2}Erf\left(\frac{x-\mu }{\sqrt{2}\sigma }\right),$$

where $\Phi \left(x_{k}\;|\;\mu ,\sigma \right)$ is the Gaussian CDF with parameters (μ,σ). The beauty of the proposed virtual combined histogram scheme is that during the luminance modification process, only the global (and local) feature sets are used. Actually, the construction of an entire histogram is not needed.

Moreover, the Gauss error function Erf(x) can be approximated from [24] as the following:

(24) $$\tanh\left(\frac{77x}{75}+\left(\frac{116}{25}\right)\tanh\left(\frac{147x}{73}-\left(\frac{76}{7}\right)\tanh\left(\frac{51x}{278}\right)\right)\right),$$

where tanh is the hyperbolic tangent function. Finally, the output reproduced LDR image was obtained from restoring the color information by the following:

(25) $$LDR_{R,G,B}\left(i,j\right)={\left(\frac{HDR_{R,G,B}\left(i,j\right)}{L_{in}\left(i,j\right)}\right)}^{s}\cdot L_{out}\left(i,j\right),$$

where $HDR_{R,G,B}$ represents the three channel values of the original HDR image, $L_{in}$ and $L_{out}$ represent the luminance before and after the proposed method, and s is the saturation factor (set as 0.6 in this study).

## 4. Experimental Results

In this section, we subjectively and objectively compare the effectiveness of the proposed method with those of the other photographic reproduction methods to confirm whether it affords more advantages than these methods. We selected seven classical and state-of-the-art methods for our experiments, including the following global-based reproduction method: Reinhard et al. [4] (published in 2005); the following three local-based reproduction methods: Ahn et al. [9] (published in 2013), Li et al. [13] (published in 2018), and Gao et al. [25] (published in 2020); and the following three decomposition-based reproduction methods: Gu et al. [14] (published in 2013), Liang et al. [17] (published in 2018), and Miao et al. [18] (published in 2019). For a comparison of the computational complexity, taking the image memorial (with size of 768 × 512) as an example, the processing time needed to generate a reproduced image is 0.252 s (in [4]), 0.533 s (in [9]), 5.301 s (in [13]), 0.627 s (in [25]), 0.788 s (in [14]), 2.189 s (in [17]), 0.733 s (in [18]), and 2.201 s (in the proposed method). All the methods are adjusted with the default parameters based on the suggestion of the original papers. In addition, the software and CPU are MATLAB R2016a and Intel Core i7, respectively.

### 4.1. Subjective Analysis

**Figure 6:**
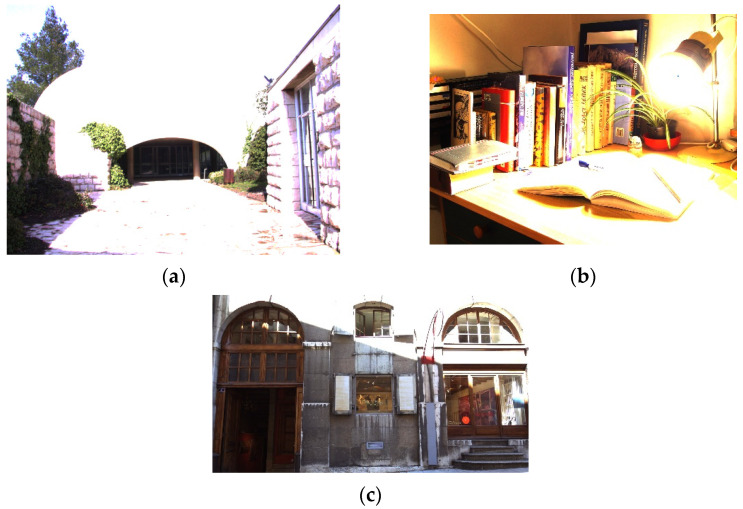
Baseline LDR images (processed using a simple linear compression), which illustrate the difficulty of photographic reproduction and can be compared with the results shown in Figure 7, Figure 8 and Figure 9. (**a**) Synagoguei. (**b**) Cadik_Desk02. (**c**) C33_Store.

In subjective analysis, the performance of different methods can be judged through side-by-side visual comparison, such as according to the amount of regional detail information, the naturalness, etc. The simple baseline LDR images shown in Figure 6 indicate that a large luminance difference exists between the highlight and shadow areas of these test images; thus, many details are lost.

**Figure 7:**
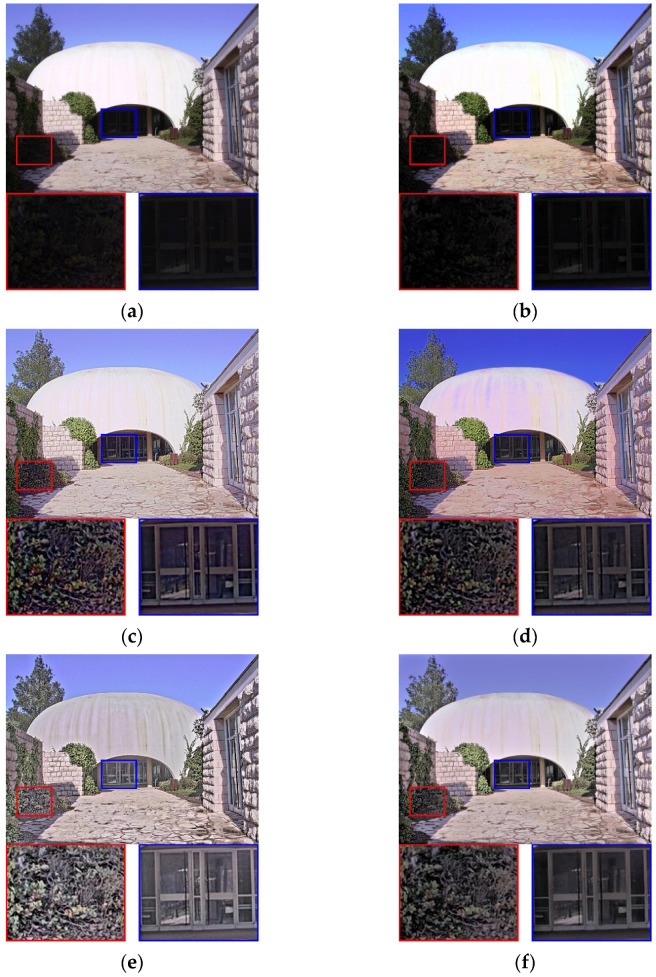

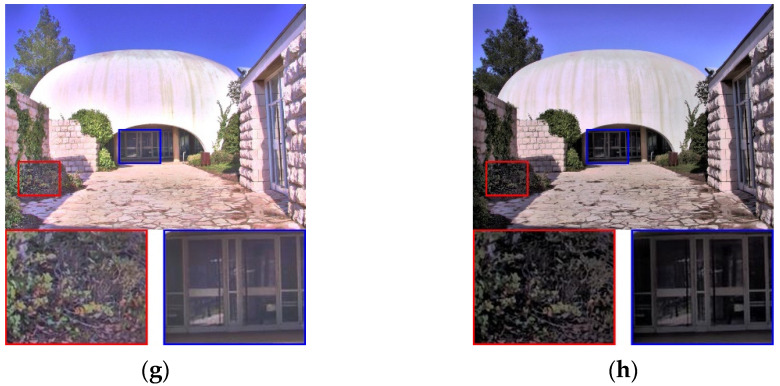
Visual comparison using the test image Synagoguei. (**a**) Result of [4]. (**b**) Result of [9]. (**c**) Result of [13]. (**d**) Result of [25]. (**e**) Result of [14]. (**f**)Result of [17]. (**g**) Result of [18]. (**h**) Result of the proposed method.

Figure 7 shows the reproduced results obtained using the Synagoguei test image. In Figure 7a, although the global brightness is balanced, the appearance of details is restricted by the global-based model. In Figure 7b, the regional scene performances in both the red and blue rectangles are poor and indistinct for the human eyes. In Figure 7c, the details of the shadow areas are preserved, whereas those of the bright area (such as the white dome) are almost imperceptible, and the tone of the entire image is monotonous and flat. In Figure 7d, the details of the red and blue rectangles are visible; however, the color of sky is oversaturated, resulting in a poor visual experience. In Figure 7e,f, although the details of the red and blue rectangles can be clearly seen, the naturalness is inevitably lost. As such methods are based on detail and base layer decomposition, image information tends to be overemphasized during decomposition and merging procedures. In Figure 7g, the details of the shadow areas (red and blue rectangles) are clear. However, the global contrast is unnatural: the highlight sky region is darkened, whereas the shadow areas are brightened, thus degrading the overall visual quality. In Figure 7h, our method shows advantages in preserving the details of the highlight and shadow areas because the proposed virtual combined histogram increases the pixel weights of local features for the highlight and shadow areas.

**Figure 8:**
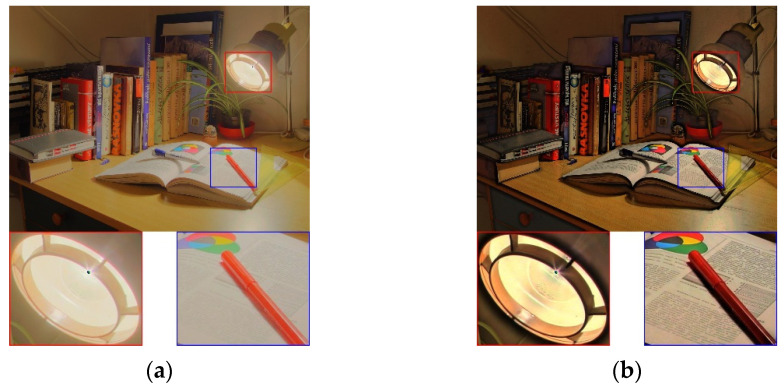

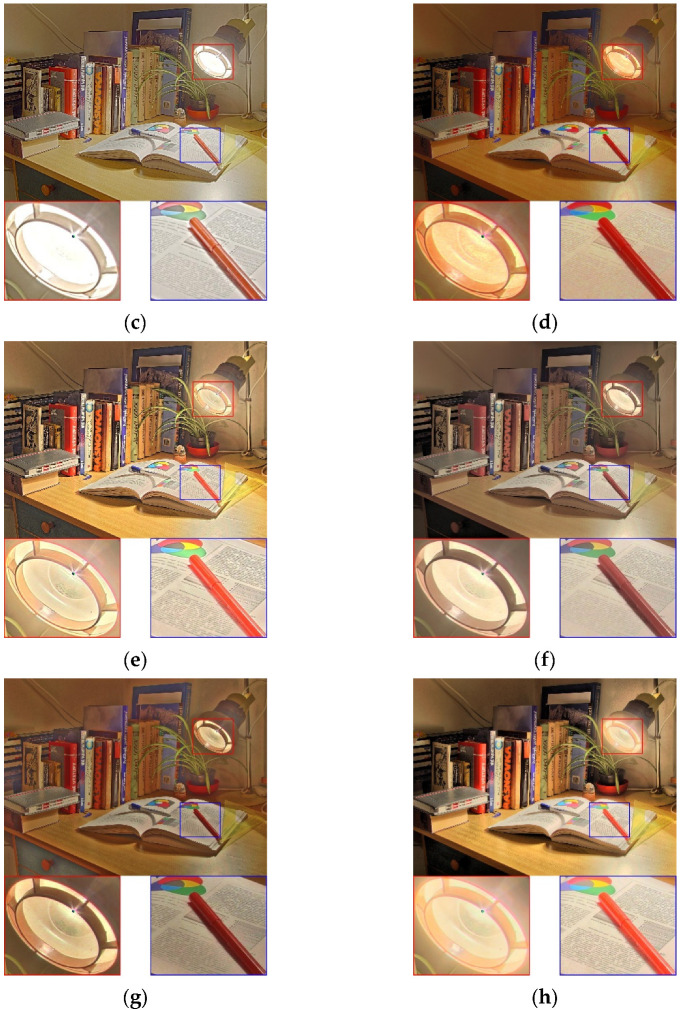
Visual comparison using the test image Cadik_Desk02. (**a**) Result of [4]. (**b**) Result of [9]. (**c**) Result of [13]. (**d**) Result of [25]. (**e**) Result of [14]. (**f**) Result of [17]. (**g**) Result of [18]. (**h**) Result of the proposed method.

Figure 8 shows the reproduced results obtained using the Cadik_Desk02 test image. It is an indoor scene in which the lamp causes an extreme luminance difference in the captured image. In Figure 8a, the text on the book is barely perceptible because of the strong lighting. In Figure 8b,c, the detailed texture of the book is maintained; however, the global contrast in both figures is unbalanced, and the color tone is flat. In Figure 8d, the details are slightly preserved; however, some color shading occurs. In Figure 8e, the details are well retained; however, the overall appearance is over-sharpened (e.g., lampshade in red rectangle). This is because in the method of [14], the detail layer and base layer are processed separately, thereby overamplifying the detail information. In Figure 8f, the details are not evident (blue rectangle), and the global contrast is insufficient. In Figure 8g, although details are visible, the overall image appears unreal owing to the imbalance between the macro- and the micro-models. In Figure 8h, our method exhibits excellent naturalness of the image. Furthermore, because of the improved visibility of the highlight and shadow areas, more visual content is retained, and the overall color naturalness is satisfactory.

**Figure 9:**
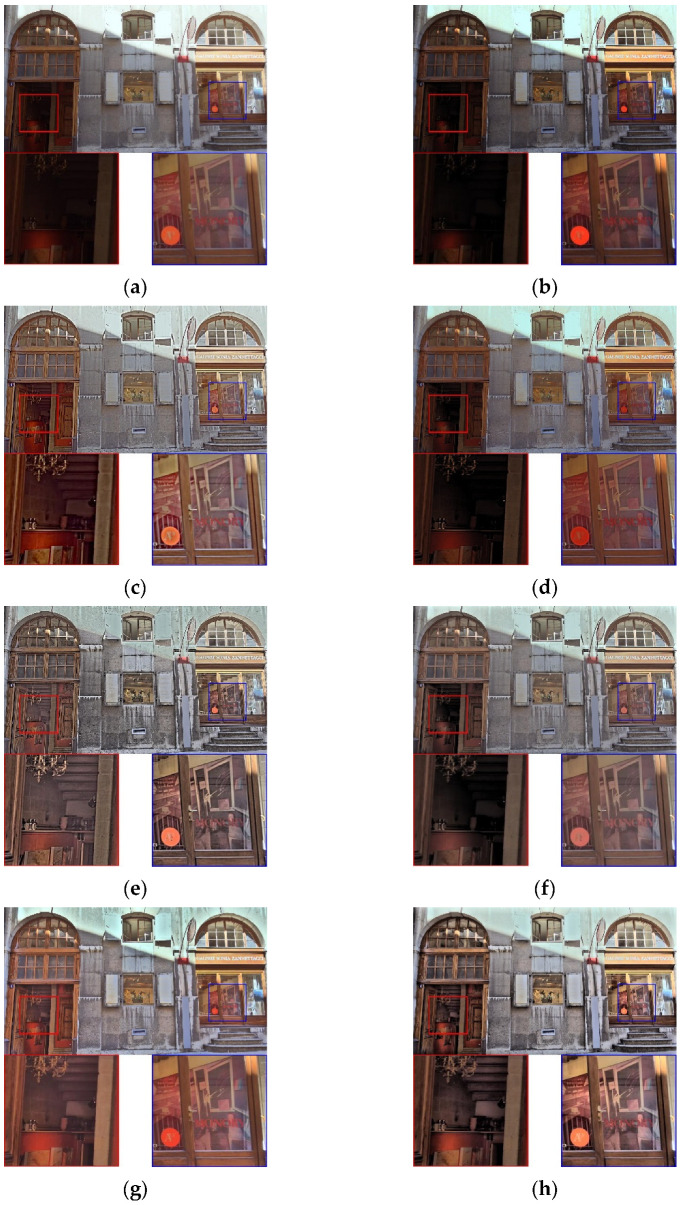
Visual comparison using the test image C33_Store. (**a**) Result of [4]. (**b**) Result of [9]. (**c**) Result of [13]. (**d**) Result of [25]. (**e**) Result of [14]. (**f**)Result of [17]. (**g**) Result of [18]. (**h**) Result of the proposed method.

Figure 9 shows the reproduced results obtained using the C33_Store test image. In Figure 9a, the detailed information of the red and blue rectangles is lost as a result of global-based processing. In Figure 9b, although the details on the right side are more visible than those in Figure 9a, the regional details of the red rectangle are lost as a result of insufficient brightness. In Figure 9c,e, detailed information is perceptible but the degree of naturalness is low and the visual effects are not rich enough. In Figure 9e, enhanced smoothing is performed without consideration of the spatial correlation of the detail layer, leading to sharper and less-natural images. In Figure 9d, the detailed information of the red rectangle is slightly visible; however, the color is not vivid enough and lacks global contrast. In Figure 9f, although the overall appearance is natural, the visibility and sharpness in the red and blue rectangles areas are insufficient. In Figure 9g, the global contrast is good and the details of the highlight (i.e., blue rectangle) and shadow (i.e., red rectangle) areas are visible; nevertheless, the image is somewhat unnatural due to the lack of global contrast. In Figure 9h, our method demonstrates favorable visual richness because both the global and local characteristics are simultaneously considered through the construction of a virtual combined histogram. Consequently, the details in the highlight and shadow areas are clearly presented and the contrast and color naturalness of the entire image are improved.

### 4.2. Objective Analysis

In addition to the described subjective analysis, several objective quality indices were also applied to evaluate whether our method outperforms the other algorithms. The first quality index is called the tone-mapped image quality index (TMQI) [26]. The TMQI evaluates the quality of the reproduced images in terms of the following three aspects: structural similarity (TMQI-S), naturalness (TMQI-N), and overall quality (TMQI-Q) as follows. The TMQI-S value can be expressed by the following:

(26) $$S=\frac{2\sigma_{x}\cdot \sigma_{y}+C_{1}}{\sigma_{x}^{2}+\sigma_{y}^{2}+C_{1}}\cdot \frac{\sigma_{xy}+C_{2}}{\sigma_{x}\cdot \sigma_{y}+C_{2}}\;,$$

where $\sigma_{x}$, $\sigma_{y}$, and $\sigma_{xy}$ are the local standard deviations and the cross-correlation between the corresponding HDR and LDR patches; and $C_{1}$ and $C_{2}$ are the positive stabilizing constants. As suggested in [26], the local window size is set as 11×11. The TMQI-N value can be expressed by the following:

(27) $$N=P_{m}P_{d}/\rho ,$$

where ρ is a normalization factor, and $P_{m}$ and $P_{d}$ are the Gaussian and the Beta probability density functions, respectively. The TMQI-Q value can be expressed by the following:

(28) $$Q=a\cdot S^{\alpha }+\left(1-a\right)\cdot N^{\beta },$$

where a is a weighting used to adjust the relative importance of the two terms (a is set as 0.8011, as suggested in [26]); S and N indicate TMQI-S and TMQI-N values, respectively; and α and β indicate their sensitivities (α=0.3046 and β=0.7088, as suggested in [26]).

As shown in Equation (26), the TMQI-S is calculated using the standard deviations and cross-correlation between the HDR images and the reproduced results. As shown in Equation (27), the TMQI-N is calculated using Gaussian and Beta probability density functions that model the histograms of the means and standard deviations in the statistics conducted on massive natural images. As shown in Equation (28), the TMQI-Q is obtained from the weighted indices of structural similarity (S value) and naturalness (N value) by using a power function to adjust these two indicators. For the TMQI-S, the TMQI-N, and the TMQI-Q, a larger index value indicates a better quality of the reproduced result. Table 1 lists the results of comparisons using Figure 7, Figure 8 and Figure 9; apparently, the proposed method not only generates more visually pleasing reproduced results (as shown in Figure 7, Figure 8 and Figure 9), but also outperforms the other seven algorithms in terms of the average TMQI-S, TMQI-N, and TMQI-Q.

**Figure 10:**
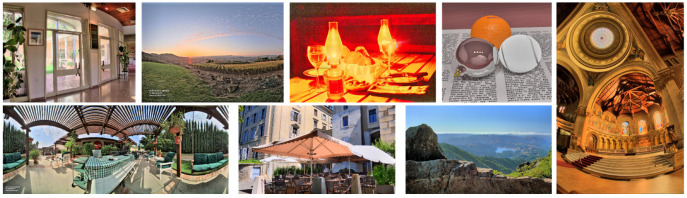
Thumbnails of partial test images with corresponding information provided in Table 2. First row from left to right: test images no. 1, no. 4, no. 6, and no. 5. Second row from left to right: test images no. 21, no. 17, and no. 3. Right side: test images no. 22. All the images are processed using the proposed method.

To further evaluate whether the proposed method is more effective than the other methods, we selected twenty-two test images from the datasets online [27,28,29,30]. Figure 10 shows some thumbnails of the test images, and Table 2 lists their names with the corresponding dynamic ranges. Moreover, four more objective quality metrics were added for conducting a thorough discussion. The first one was the feature similarity index for tone-mapped images (FSITM-TMQI) [31], an improved version of the TMQI that is based on a comparison of the phase-derived feature maps of the original HDR and the reproduced images. As in the case of the TMQI, a larger FSITM-TMQI value indicates a higher image quality. The second one was the dubbed blind/referenceless image spatial quality evaluator (BRISQUE) [32]. Unlike the TMQI and FSITM-TMQI, the BRISQUE is a no-reference quality assessment that evaluates the possible loss of naturalness in the spatial domain through scene statistics. The third one is the Blind TMQI (BTMQI) [33], another type of no-reference quality assessment that evaluates image quality by introducing features of statistical naturalness, structural preservation, and information entropy. For both the BRISQUE and BTMQI, lower values indicate less loss of overall naturalness, that is, better quality. The fourth one is the Integrated Local Natural Image Quality Evaluator (IL-NIQE) [34], which is a non-reference quality evaluation based on integrating multiple image statistics such as texture, color, and contrast. The IL-NIQE value reflects the global naturalness of the output image. The lower the IL-NIQE value is, the more natural it is.

**Figure 11:**
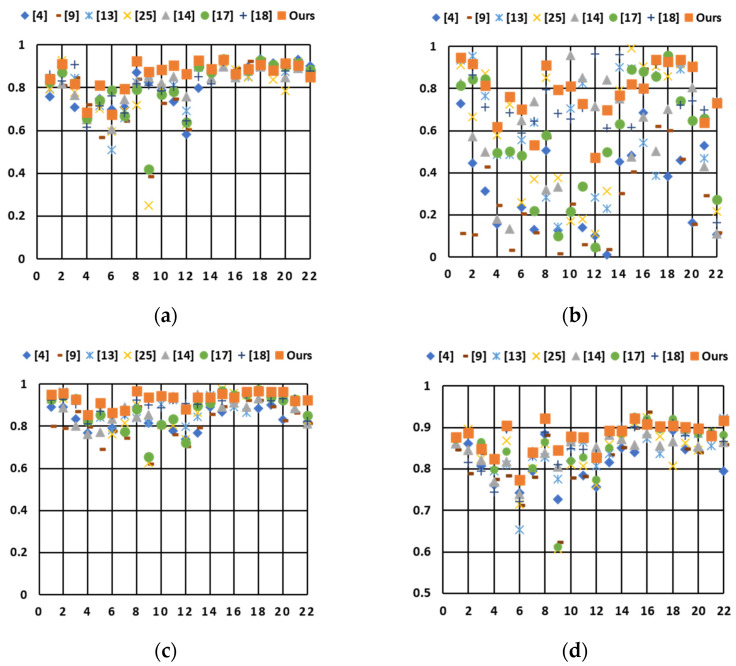

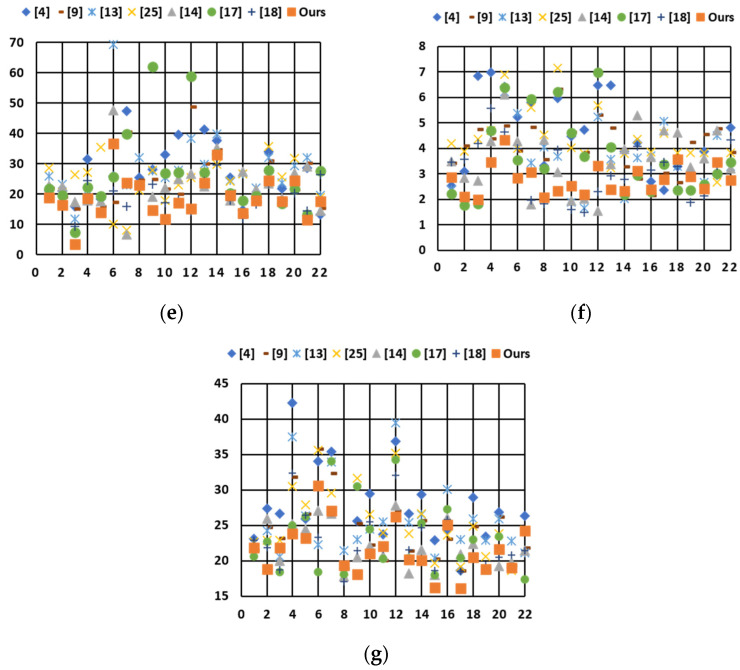
Comparison of scatter plots using the twenty-two test images, where the horizontal axis indicates the image order, and the vertical axis indicates the objective quality index. (**a**) Result of TMQI-S. (**b**) Result of TMQI-N. (**c**) Result of TMQI-Q. (**d**) Result of FSITM-TMQI. (**e**) Result of BRISQUE. (**f**) Result of BTMQI. (**g**) Result of IL-NIQE.

The scatter plot in Figure 11 shows the detailed information of all twenty-two test images with each of the seven objective quality indices—TMQI-S, TMQI-N, TMQI-Q, FSITM-TMQI, BRISQUE, BTMQI, and IL-NIQE. This figure shows that the performance of the proposed method was among the top three for most evaluation indicators. Table 3 lists the average scores of the twenty-two test images obtained using different methods. With regard to the full-reference quality assessments (TMQI-S, TMQI-N, TMQI-Q, and FSITM-TMQI), our method obtained the best scores for these four assessments. The proposed method achieved the highest scores for the average TMQI-S, TMQI-N, and TMQI-Q, indicating that it achieved a strong balance between image structure and naturalness. In addition, our method also obtained the highest score for the average FSITM-TMQI, indicating that it generated more visually pleasing images based on the evaluation using phase-derived feature maps.

With regard to the no-reference quality assessments (BRISQUE, BTMQI, and IL-NIQE), our methods all obtained the best scores of the average BRISQUE, BTMQI, and IL-NIQE. By considering both global and local features to generate a virtual combined histogram, this method maintains the naturalness of an image and produces an output reproduced image with high image quality. Compared with global- and local-based reproduction methods that consider only global features (or only local features), our method can simultaneously take advantage of global and local features. Compared with the decomposition-based methods, our method does not need to process the base and detail layers separately, thus avoiding unnaturalness when blending different image layers. Overall, in Table 3, our method achieved the highest score in all seven assessments, indicating its excellent performance with natural-looking and rich information.

**Figure 12:**
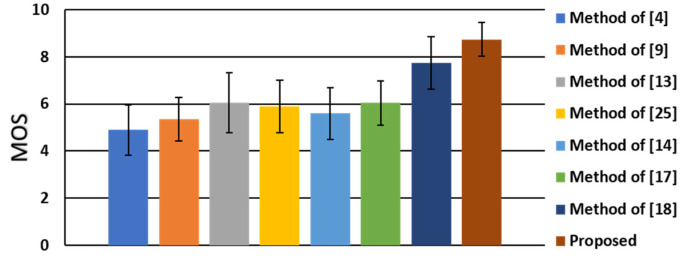
Mean and standard deviation of subjective rankings of the eight comparative algorithms.

For the subjective analysis and evaluation, we invited 20 participants (10 males and 10 females) to take a subjective visual quality test. The participants were asked to rate the visual subjectiveness of all the images without knowing the applied method on the output images of twenty-two scenes using the eight comparative algorithms. The score ranges from 1 to 10 points, where 1 point means “unsatisfied” and 10 points means “excellent”. The mean and standard deviation of the mean opinion scores (MOS) of the subjective users are shown in Figure 12, where the proposed method is significantly better than the other methods.

**Figure 13:**
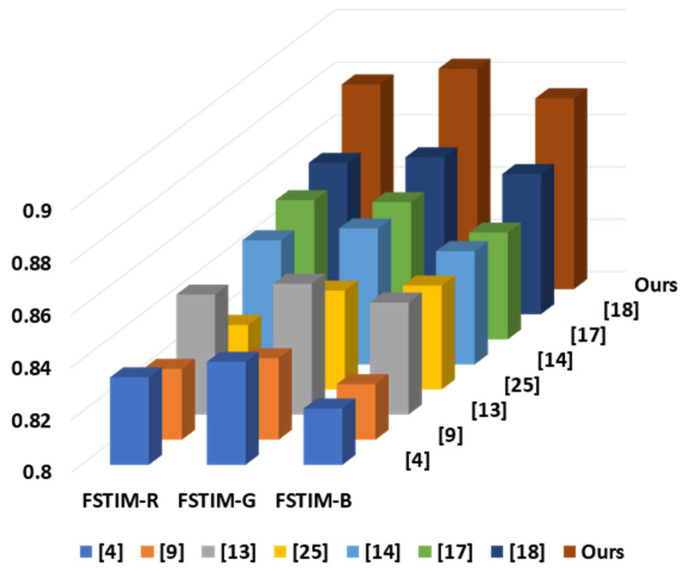
Comparison of the average FSITM-R, FSITM-G, and FSITM-B using the twenty-two test images.

In addition, the abovementioned FSITM-TMQI is actually obtained by averaging the scores of RGB channels, i.e., the FSITM-R, FSITM-G, and FSITM-B, respectively. The FSITM quality evaluation index is based on using the local phase similarity to construct a noise-independent feature map in the R, G, and B planes. In view of this, we further compare the average FSITM-R, FSITM-G, and FSITM-B using the twenty-two test images. As shown in Figure 13, our improved method performs better than the other seven reproduction methods in all the RGB channels of the FSITM, indicating that our method is not only really close to the real-world scene but also has an attractive visually pleasing character and natural color appearance.

## 5. Conclusions

Although HDR cameras are popularized in the digital photography industry, the current price of an HDR display is unaffordable to common people. Therefore, photographic reproduction techniques have great commercial potential due to the limited availability of HDR displays. This paper presented a new reproduction method, which considers global/local features simultaneously to achieve both global contrast-maintenance and local detail-preservation. Instead of performing the global-based and local-based processes separately, we combined two statistical approaches to extract the mutually compatible features to form a virtual combined histogram. In the feature fusion stage, a weight map is used to modify the importance between the global and local features. Moreover, with the integration of Gauss error function and global/local feature sets, the construction of an entire histogram is not actually needed in the luminance modification stage. From the experimental results, the proposed method outperforms other state-of-the-art methods in terms of various visual comparisons (Figure 7, Figure 8 and Figure 9) and objective evaluations (Table 1 and Table 3, Figure 11 and Figure 13). In the future, we plan to conduct the Wilcoxon test and the Friedman test to check whether the experimental results are statistically significant.

## Figures and Tables

**Table 1:** Comparison of TMQI-S, TMQI-N, and TMQI-Q using the test images shown in Figure 7, Figure 8 and Figure 9.

**TMQI-S (Structural Similarity)**								
**Method**	[4]	[9]	[13]	[25]	[14]	[17]	[18]	**Ours**
Synagoguei	0.8724	0.8414	0.8254	0.7177	0.8266	0.7906	0.8191	0.9241
Cadik_Desk02	0.7338	0.7465	0.8116	0.7883	0.8516	0.7815	0.8037	0.9049
C33_Store	0.9342	0.9326	0.8924	0.9235	0.8972	0.9255	0.9090	0.9072
**Average**	**0.8468**	**0.8402**	**0.8431**	**0.8098**	**0.8584**	**0.8326**	**0.8439**	**0.9121**
**TMQI-N (Naturalness)**								
Synagoguei	0.5045	0.5690	0.2826	0.8492	0.3186	0.5785	0.7930	0.9113
Cadik_Desk02	0.1387	0.0615	0.8236	0.1790	0.8517	0.3349	0.7084	0.7269
C33_Store	0.3809	0.6014	0.9239	0.8563	0.6999	0.9555	0.9104	0.9278
**Average**	**0.3414**	**0.4106**	**0.6767**	**0.6282**	**0.6234**	**0.6230**	**0.8039**	**0.8554**
**TMQI-Q (Overall Quality)**								
Synagoguei	0.8910	0.8934	0.8369	0.9013	0.8444	0.8808	0.9226	0.9683
Cadik_Desk02	0.7781	0.7605	0.9251	0.8039	0.9403	0.8348	0.9251	0.9358
C33_Store	0.8851	0.9230	0.9633	0.9601	0.9295	0.9750	0.9642	0.9663
**Average**	**0.8514**	**0.8590**	**0.9084**	**0.8884**	**0.9048**	**0.8969**	**0.9373**	**0.9568**

**Table 2:** List of twenty-two test images and their dynamic ranges (D).

No.	Name	D	No.	Name	D
1	Belgium	5.87	12	Cadik_Window	5.10
2	Fop_map	4.12	13	C19_Casement	2.46
3	Mt. Tam West	4.06	14	C21_Studio	2.88
4	Napa_Valley	5.36	15	C22_Fort	2.79
5	Rend01	5.84	16	C29_Buildings	3.52
6	Still_Life	3.91	17	C31_Parasol	3.57
7	Spheron_Siggraph	5.01	18	C33_Store	2.57
8	Synagogue	2.58	19	C37_Sculptures	4.17
9	Design Center	5.25	20	C38_Cross	3.65
10	Cadik_Desk01	5.68	21	Spheron_PriceWestern	3.73
11	Cadik_Desk02	4.26	22	Memorial	5.53

**Table 3:** Overall comparison of average TMQI, FSITM-TMQI, BRISQUE, BTMQI, and IL-NIQE using the twenty-two test images.

Method	[4]	[9]	[13]	[25]	[14]	[17]	[18]	Ours
**TMQI-S**	0.8144	0.7946	0.8085	0.7737	0.8197	0.8066	**0.8199**	**0.8606**
**TMQI-N**	0.3631	0.2765	0.6334	0.6143	0.5898	0.5689	**0.7258**	**0.7805**
**TMQI-Q**	0.8464	0.8185	0.8906	0.8734	0.8838	0.8776	**0.9046**	**0.9308**
**FSITM-TMQI**	0.8314	0.8265	0.8462	0.8340	0.8475	0.8487	**0.8571**	**0.8784**
**BRISQUE**	28.9897	23.6616	28.2828	23.6827	22.9164	26.2905	**18.9477**	**18.9413**
**BTMQI**	4.5153	4.0962	3.4366	4.3851	3.5776	3.6170	**3.1737**	**2.7792**
**IL-NIQE**	27.0699	24.0626	25.6440	25.1176	**22.0920**	22.9561	22.8037	**21.6186**

## Data Availability

Not applicable.
